# Underwater Endoscopic Submucosal Dissection via Continuous Irrigation Method for a Colorectal Tumor Involving the Appendiceal Orifice

**DOI:** 10.1111/den.70071

**Published:** 2025-11-30

**Authors:** Takahiro Muramatsu, Masakatsu Fukuzawa, Takao Itoi

**Affiliations:** ^1^ Department of Gastroenterology and Hepatology Tokyo Medical University Hospital Tokyo Japan

## Abstract

Watch a video of this article.

## 1 Brief Explanation

**FIGURE 1:**
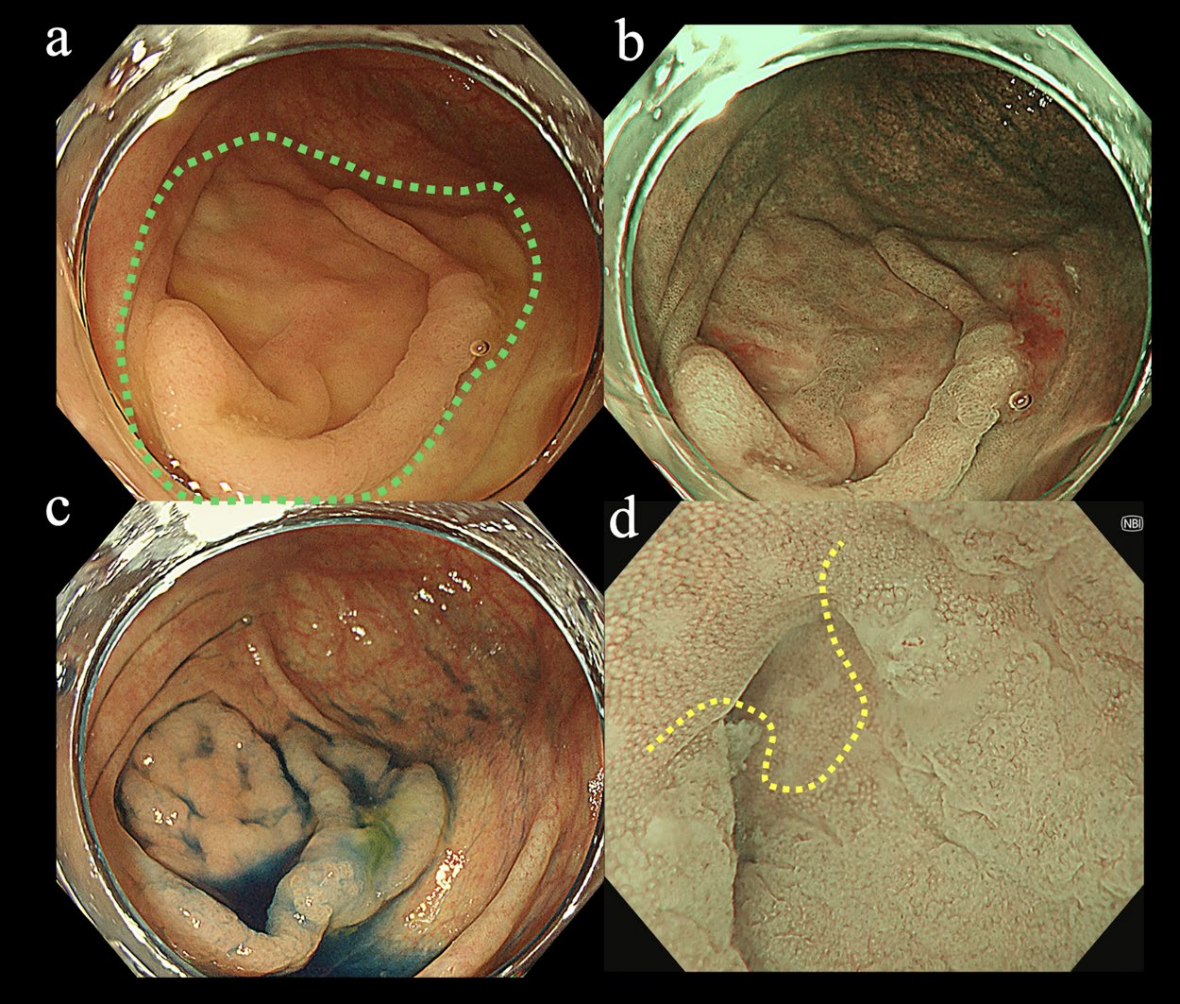
Endoscopic images. (a) White‐light image. Lower gastrointestinal endoscopy revealed a flat, isochromatic lesion (35 mm, type 0‐IIa) in the cecum. The boundary of the lesion is indicated by the green dotted line. (b) Narrow‐band imaging (NBI) view. (c) Indigo carmine dyeing clarified the boundaries of the lesion. (d) The lesion extended into the appendiceal orifice. The boundary of the lesion within the appendiceal orifice is indicated by the yellow dotted lines.

**FIGURE 2:**
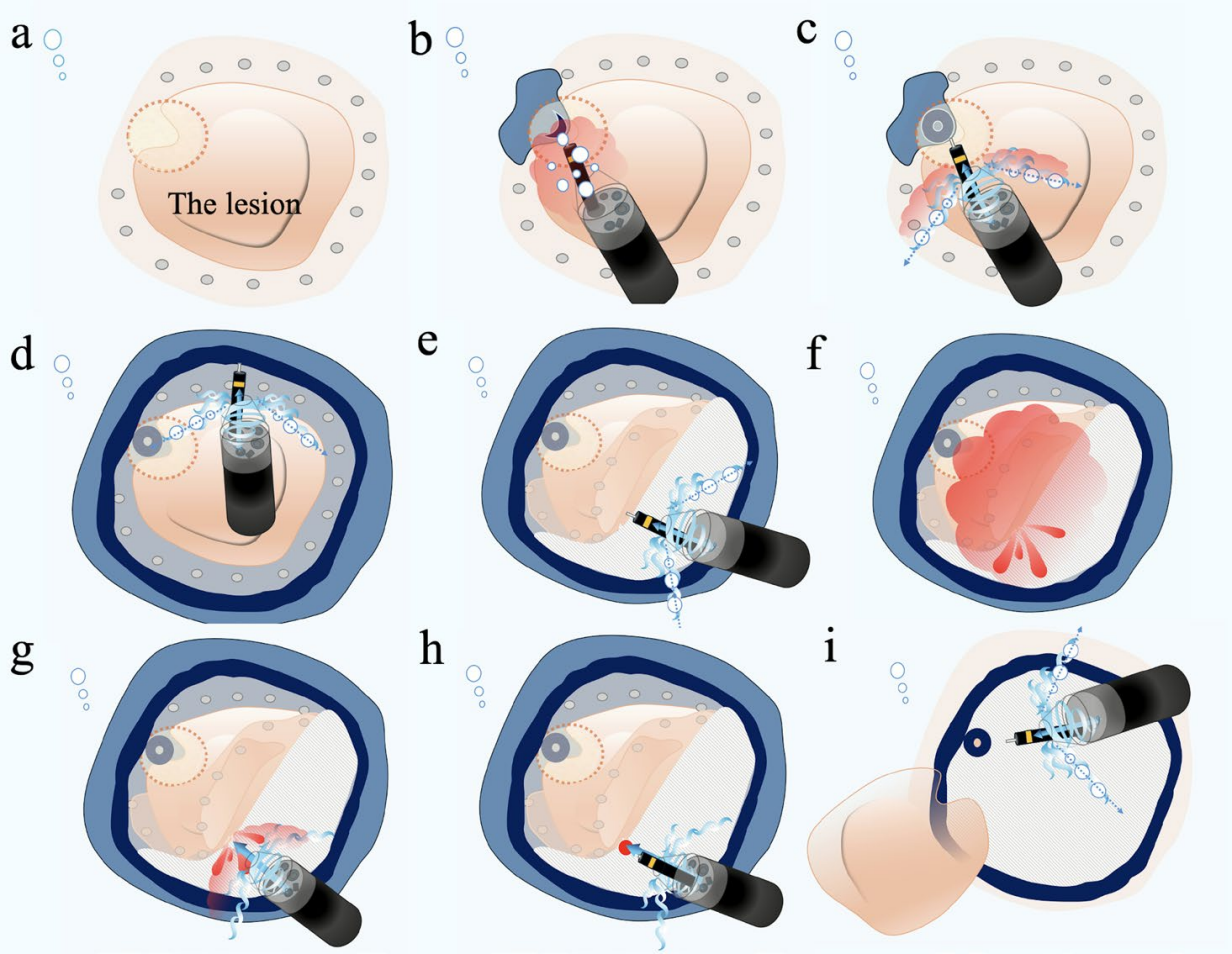
Schema of underwater endoscopic submucosal dissection using the continuous irrigation method for a colorectal tumor involving the appendiceal orifice. (a) Underwater view. Marking was performed around the lesions. The appendiceal orifice is indicated by orange dotted lines. (b) A mucosal incision was made on the appendiceal side of the lesion. Bleeding and air bubbles associated with the incision resulted in a poor endoscopic view. (c) Bleeding and bubbles were cleared using the continuous irrigation method (CIM), resulting in an improved, clear endoscopic view. (d) A complete circumferential incision was made. (e) Submucosal dissection was performed using CIM. (f) Bleeding occurred. (g) CIM helped secure the endoscopic view and provided a tamponade effect, facilitating easier identification of the bleeding point. (h) Complete hemostasis was achieved using CIM. (i) Complete en bloc resection was successfully achieved.

Endoscopic submucosal dissection (ESD) for lesions extending into the appendiceal orifice remains technically challenging due to limited accessibility and risks of perforation or appendicitis [1]. Although techniques such as traction methods [2] and tapered hoods [3] have been reported, a standardized strategy has not yet been established. Herein, we report successful underwater ESD (UESD) [4] for a colorectal tumor involving the appendiceal orifice using the continuous irrigation method (CIM) [5], which provides improved visualization and facilitates precise mucosal incision and submucosal dissection by generating water pressure (Video S1). A 47‐year‐old woman underwent colonoscopy following positive fecal occult blood test results. The procedure revealed a flat, isochromatic lesion measuring 35 mm (type 0‐IIa) in the cecum (Figure 1a–c). The lesion was suspected to be a sessile serrated lesion (SSL) and extended into the appendiceal orifice (Figure 1d). ESD was planned using a small‐caliber tapered conical hood to improve access to the appendiceal orifice. After lesion marking (Figure 2a), mucosal incision began on the appendiceal side. However, bleeding and bubbles obscured the endoscopic view; therefore, CIM was initiated. CIM rapidly cleared the visual field and utilized water pressure to effectively separate the lesion from the appendiceal orifice, facilitating accurate incision (Figure 2b–d). Circumferential incision and subsequent submucosal dissection were successfully completed under stable visualization provided by CIM (Figure 2e–l). The procedure lasted 91 min.

The pathological diagnosis was SSL. CIM is a simple approach that improves visualization by clearing air bubbles and bleeding during UESD. Additionally, CIM supports mucosal incision and submucosal dissection through the water pressure effect and facilitates hemostasis via temporary compression, thus enabling safe and efficient en bloc resection of lesions involving the appendiceal orifice.

## Author Contributions

Takahiro Muramatsu wrote and edited the manuscript. Takahiro Muramatsu approved the manuscript. Takahiro Muramatsu, Masakatsu Fukuzawa, and Takao Itoi reviewed the literature and revised the manuscript for intellectual content.

## Funding

The authors received no specific funding for this work.

## Consent

Informed consent was obtained from the patient for the publication of this report.

## Conflicts of Interest

The authors declare no conflicts of interest.

## Supporting information


**Video S1:** UESD via CIM for a tumor involving the appendiceal orifice.
